# Liver cancer knowledge graph construction based on dynamic entity replacement and masking strategies RoBERTa-wwm-large-BiLSTM-CRF model with clinical Chinese EMRs

**DOI:** 10.3389/frai.2025.1663877

**Published:** 2025-10-17

**Authors:** Yichi Zhang, Xiaojun Hu, Hailing Wang, Ke Liu, Yongbin Gao, Xiaoyan Jiang, Yingfang Fan, Zhijun Fang

**Affiliations:** ^1^School of Electronic and Electrical Engineering, Shanghai University of Engineering Science, Shanghai, China; ^2^The Department of Hepatobiliary Surgery, Southern Medical University Third Hospital, Guangzhou, China; ^3^Beijing Anding Hospital, Capital Medical University, Beijing, China; ^4^School of Computer Science and Technology, Donghua University, Shanghai, China

**Keywords:** knowledge graph, named entity recognition, liver cancer, knowledge fusion, knowledge graph application

## Abstract

**Introduction:**

Liver cancer is a leading cause of cancer-related mortality worldwide, necessitating advanced tools for diagnosis and management. Knowledge graphs (KGs) are crucial for advancing smart healthcare, but existing liver cancer-specific KGs are mostly derived from literature or public databases, lacking integration with real-world clinical data [e.g., Electronic Medical Records (EMRs)], creating a critical gap. Furthermore, there is currently no publicly available KGs specifically for liver cancer, creating a significant gap in structured clinical knowledge resources.

**Methods:**

This study proposes a novel framework to construct the first Chinese liver cancer KG from Real-World Liver Cancer Electronic Medical Records (RLC-EMRs). A new named entity recognition (NER) model, DERM-RoBERTa-wwm-large-BiLSTM-CRF was developed that uses a Dynamic Entity Replacement and Masking (DERM) strategy to address data scarcity. Knowledge fusion was performed using the TF-IDF algorithm to standardize and integrate entities from clinical records, the professional medical website www.XYWY.com, and the CCMT-2019 terminology standard.

**Results:**

The final constructed liver cancer KG contained 46,364 entities and 296,655 semantic relationships. The proposed NER model achieved a state-of-the-art F1 score of 68.84% on the public CMeEE-v2 dataset. On the proprietary RLC-EMRs dataset, the model demonstrated high effectiveness with a precision of 93.23%, recall of 94.69%, and an F1 score of 93.96%. In addition, a KG-based retrieval system was successfully developed to query for complications, medications, and other related information.

**Discussion:**

The findings demonstrated the effectiveness of the proposed framework in constructing a comprehensive and clinically relevant liver cancer KG. The novel DERM-based NER model significantly improved entity extraction from complex medical texts. By successfully integrating real-world clinical data, this study addresses a critical gap in existing liver cancer-specific KGs, which are mostly derived from literature or public databases and lack integration with real-world clinical information.

## 1 Introduction

The 2025 American Cancer Society estimates that liver cancer accounts for approximately 42,240 new cases (2.07% of all cancers), ranking 14th among major cancer types (Siegel et al., 2025). Furthermore, liver cancer accounts for 4.87% of all cancer-related fatalities, equivalent to approximately 30,090 deaths, ranking 6th in terms of cancer mortality. Particularly in China, liver cancer remains a major burden, ranking fourth in new cancer cases nationwide and second in cancer-related deaths, reflecting ongoing challenges in both incidence and mortality rates despite national prevention efforts (Chinese Society of Liver Cancer, 2025). The most common type of primary liver cancer is hepatocellular carcinoma (HCC), which accounts for 75–85% of (Bray et al., 2024). Postsurgical complications of liver cancer include infection, bleeding, liver failure, and various systemic complications. These complications significantly affect patient outcomes and quality of life, with some being potentially life-threatening. The complexity of liver cancer management and its associated complications necessitate a comprehensive understanding of the risk factors, treatment outcomes, and potential complications. Hence, the development of an evidence-based knowledge graph (KG) can provide healthcare providers with a sophisticated tool for visualizing and analyzing the intricate relationships between various risk factors, complications, and treatment (Abu-Salih et al., 2023).

The KG was first proposed by Google in 2012 as a structured knowledge representation of real-world entities (e.g., people, address, events, etc.) and the relationships between them (e.g., “lives in,” “works at,” “has,” etc.) as graphical structures. Since then, KG has found extensive applications (Wang et al., 2020) representation method that encodes entities (e.g., diseases, medications, symptoms, and operations) and relationships between entities (e.g., drug-disease treatment relationships, disease-symptom association relationships, etc.) in the medical domain as structured (Chen et al., 2019). Through the construction of a medical KG, it is possible to efficiently organize, retrieve, and reason medical knowledge, thereby facilitating applications such as clinical decision support, medication recommendations, and disease forecasting.

KG also uses visualization techniques to show how different pieces of knowledge and their connections look. Specifically, it organizes and represents knowledge using sets of “triples” that consist of a subject, relationship, and object (Ferrucci et al., 2013). These triples show different entities, and how they relate to each other in each domain, where each triple is called a fact. In a KG, nodes represent entities and edges illustrate the relationships between them. For example, “liver cancer” can be treated as a subject node, “right upper abdominal pain” can be treated as an object node, and the relationship between “liver cancer” and “right upper abdominal pain” is the “symptom” which can be treated as an edge.

The construction of healthcare KGs is an active research area, with many KGs being built from biomedical literature and public databases (Cui et al., 2025). For instance, large-scale KGs like the Unified Medical Language System (UMLS) (Bodenreider, 2004) and SemMedDB (Kilicoglu et al., 2012) provide broad-spectrum medical knowledge by extracting relationships from scientific publications. While incredibly valuable, these general biomedical knowledge graphs are often too broad to capture the details required for specific, complex diseases like liver cancer and lack the patient-specific information found in clinical practice (Al Khatib et al., 2024). Current research on medical KG construction predominantly relies on medical literature and professional websites as data sources, particularly in specialized disease domains such as diabetes (Wang et al., 2020) and COVID-19 (Chatterjee et al., 2021). Recent advancements in liver cancer-specific applications include graph-based approaches for ontology enrichment and link prediction (Essalah et al., 2024) and reviews of graph theory in liver disease research (Hu et al., 2025). However, these works notably lack integration with real clinical data. Specifically, systematic KG research has been absent from the existing article on liver cancer. Therefore, the construction of a comprehensive liver cancer KG that incorporates real clinical data remains an urgent challenge.

To address these limitations, this paper proposes an innovative framework for liver cancer KG construction. The main contributions of this study are summarized as follows:

1. This paper proposes a comprehensive framework for constructing a Chinese liver cancer KG using Real-World Liver Cancer Electronic Medical Records (RLC-EMRs). This approach addresses a critical gap by structuring unstructured clinical narratives into a KG.
2. This paper proposes a tailored NER method, DERM-RoBERTa-wwm-large-BiLSTM-CRF, which incorporates a dynamic entity replacement and masking strategy (DERM). The model significantly improves both accuracy and robustness in extracting complex medical entities from publicly dataset CMeEE-v2 and RLC-EMRs.
3. An intelligent system based on the KG is developed for multi-hop retrieving information related to liver cancer, such as complications, medications, foods, and so on related to liver cancer.

## 2 Related work

### 2.1 Medical named entity recognition

NER represents a crucial aspect of natural language processing (NLP), focusing on extracting entities with defined meanings, including diseases, symptoms, and medicines, specifically within medical literature. The evolution of deep learning methods has significantly advanced Chinese medical NER in recent years. Prior to the widespread adoption of deep learning, traditional approaches in this field predominantly utilized rule-driven and statistical methodologies. Rule-based techniques rely on predefined rules and domain-specific dictionaries for tasks like entity identification, leveraging tools such as regular expressions and dictionary lookups. Statistical models, including the Hidden Markov Model (HMM) (Morwal, 2012), Maximum Entropy Markov Model (MEMM) (Saha et al., 2009), and Conditional Random Field (CRF) (Sutton, 2012) are frequently employed. These traditional methods offered benefits such as straightforward implementation, notable accuracy, and reduced computational demands in certain scenarios. These models rely on rules and dictionaries formulated by domain experts, require a lot of human involvement, have difficulty dealing with complex and flexible linguistic phenomena, and have a weak generalization ability. With the development of word vector techniques (e.g., word2vec, Goldberg and Levy, 2014) and Glove (Pennington et al., 2014), breakthroughs have been made in the field of Chinese medical entity recognition (CMNER). Word vectors can characterize words into continuous high-dimensional vectors, thus improving the model’s ability to capture the semantics of words. Through unsupervised learning, word vectors can be learned from a large amount of unlabeled data, and the semantic relationships between words can be captured effectively. However, word vector representation is inaccurate for new words or words with multiple meanings. In recent years, deep learning techniques have been widely used in Chinese medical NER. The main methods include convolutional neural networks (CNN) (Wu et al., 2017), recurrent neural networks (RNN) (Sherstinsky, 2020), and long short-term memory networks (LSTM) (Sherstinsky, 2020). These methods can effectively capture the local features and long-distance dependencies of text to improve the accuracy of NER. Automatic learning of local features and long-distance dependencies of text can provide better modeling of complex and flexible linguistic phenomena. However, these methods require a large amount of labeled data for training, and the training process is time-consuming. With the emergence of pre-trained models, such as BERT (Devlin et al., 2019), RoBERTa (Liu et al., 2019) and GPT3 (Brown et al., 2020), Chinese medical NER research has entered a new era (Peng et al., 2023) introduces a TENER-based pre-trained model that divides the NER task into two branches: one for identifying entity boundaries and another for classifying entity types (Tang et al., 2024) combines the Segmentation Synonym Sentence Synthesis (SSSS) algorithm based on neighboring vocabulary with RoBERTa-BiLSTM-CRF. The models achieved F1 scores of 91.30 and 91.35% on the CCKS-2017 dataset.

### 2.2 Construction of medical knowledge graph

Medical KGs are characterized by dispersed knowledge distribution, distinctive syntax, and non-standardized terminology, which makes the construction of medical KGs more difficult. In response to these challenges, researchers have undertaken diverse approaches to construct a Chinese medical KG. For example, Zhang et al. (2018) proposed a generative framework known as the Conditional Relationship Variational Autoencoder, designed to streamline data preprocessing and minimize the need for manual annotation in the Chinese medical text corpus (Zhao et al., 2020). To improve NER and relation extraction tasks in Clinical Electronic Medical Records (CEMRs), researchers have leveraged advanced deep learning techniques. Sheng et al. (2019) developed a comprehensive framework for a health KG, focusing on cardiovascular disease Electronic Medical Records (EMRs). Zhou et al. (2019) investigated developing and utilizing a “knowledge-centric” traditional Chinese medicine KG derived from ancient Chinese texts. However, one-way semantic relationships are inadequate for fully representing the complexities of patient medical processes. For example, semantic links between diseases and diagnostic procedures include both identifying the illness and uncovering it through detailed medical evaluation (Li et al., 2020a; Li et al., 2020b; Li L. et al., 2020) introduces a structured methodology for building medical KGs using large-scale EMRs, resulting in a KG with nine distinct entity categories, 22,508 individual entities, and 579,094 quadruplets. Xiu et al. (2020) develops a framework aimed at constructing a KG for digestive system tumors derived from CEMRs, achieving a semantic-driven digestive system tumor knowledge graph (DSTKG). Shang et al. (2024) employed the Observational Medical Outcomes Partnership (OMOP) vocabulary and a unified semantic framework to standardize local EHR datasets for constructing KG.

Applications of medical KGs, as illustrated by the semantic web for Chinese medicine, have captured significant interest from researchers and the medical sector. Their value in smart use cases like analytical data mining and personalized drug suggestions is especially noteworthy. For example, Gong et al. (2021) introduced a framework for Safe Medicine Recommendation (SMR), framing the task as a link prediction challenge.

Our work on constructing liver cancer KG from CEMRs distinguishes itself from previous efforts in several key aspects: (1) it introduces the first KG specifically tailored for liver cancer, diverging from the general medical KGs typically seen in prior research; (2) it involves normalizing and interconnecting entities like diseases, treatments, and surgical records in CEMRs with online medical knowledge bases; and (3) adding the downstream applications of the KG, rather than focusing only on the specific steps of construction KG as in previous work.

## 3 Method

**Figure 1:**
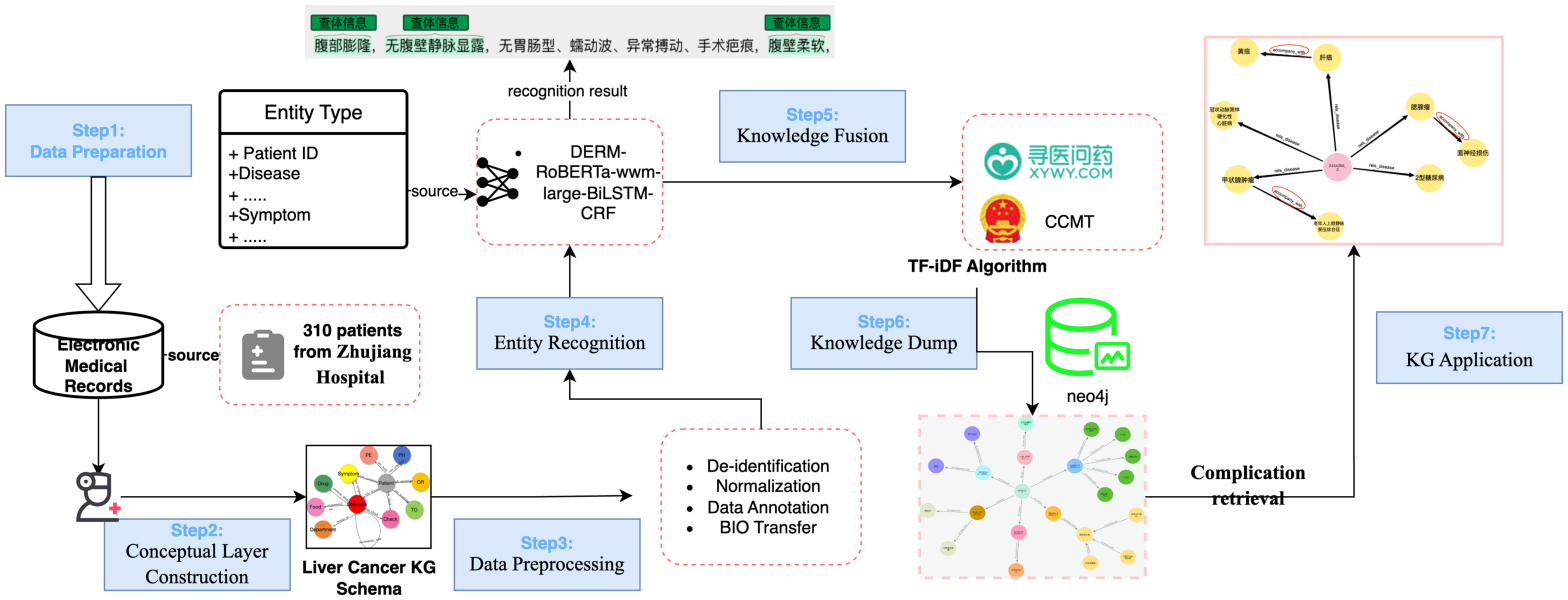
The proposed framework of liver cancer KG construction. The procedure consists of seven key steps: (1) Data Preparation, (2) Conceptual Layer Construction, (3) Data Preprocessing, (4) Entity Recognition, (5) Knowledge Fusion (KF), (6) KG construction, visualization and quality assessment, (7) KG Application. It is important to note that Steps 3 and 4 often demand extensive practical experience with Chinese EMRs and online resources.

This section outlines a structured approach to construct the liver KG from RLC-EMRs as illustrated in Figure legends (Figure 1).

### 3.1 Data preparation

The datasets used in this study include the publicly available CMeEE-v2 dataset^1^ and private dataset RLC-EMRs.

The CMeEE-v2 dataset is a widely used Chinese biomedical NER benchmark, originally introduced in the CHIP 2020 challenge under the CBLUE evaluation framework. It contains approximately 23,000 annotated medical sentences, including 15,000 for training, 5,000 for development, and 3,000 for testing, with 81,020 entity mentions. The dataset covers nine medical entity categories: diseases, symptoms, drugs, medical equipment, procedures, body parts, examination items, microorganisms, and departments.

The RLC-EMRs dataset consisted of three parts: CEMRs, the professional medical website XYWY.com, and the Clinical Chinese Medical Terminology 2019 edition (CCMT-2019). The CEMRs were provided by the Zhujiang Hospital of Southern Medical University in Guangzhou, containing EMRs of 304 liver cancer patients from 2015 to 2020. This recorded liver cancer patients’ information, including admission records, medical records, surgical records, and discharge summaries.

All patients enrolled in this study provided written informed consent upon admission, permitting the use of their clinical data for research purposes. To create a high-quality clinical corpus, we first established a set of rigorous selection criteria in collaboration with doctors from Zhujiang Hospital of Southern Medical University. For inclusion, patient EMRs were required to have a postoperative pathology report confirming a single, primary liver tumor and complete immunohistochemistry results. Conversely, records were excluded if the patient had received any form of preoperative anti-tumor treatment, including radiofrequency ablation, hepatic artery chemoembolization, targeted therapy, or immunotherapy, or if their immunohistochemistry results for CK19 were missing. This stringent selection process ensured the dataset consisted of well-documented, primary liver cancer cases, thereby minimizing potential biases from prior medical interventions or incomplete records.

XYWY.com is a public professional online Chinese website, it provides comprehensive information on various diseases, including symptoms, diagnoses, treatments, medications, food recommendations, departments, and complications. In this research, semi-structured knowledge pertinent to liver cancer was extracted, including the attributes of disease, five relationships between symptoms and disease, disease and drugs, disease and complications, disease and department, and disease and food.

The CCMT-2019 was published by the National Health Commission of the People’s Republic of China. It aims to standardize medical terms, provide standardized medical records, classify and code diseases, classify and code of surgical procedures, and standardize of medical terms. In this study, non-standard entities, such as operation recording, treatment options, disease, and symptoms in the EMRs will be aligned with the standardized entities in the CCMT-2019. Eventually, the aligned entities were fused with the entities from XYWY.com to expand the dataset.

### 3.2 Conceptual layer design

Based on the recommendations of the hospital expert and the characteristics of the RLC-EMRs dataset, 11 types of Liver cancer entities were defined in this study, including patient, examination, symptom, diseases, past history (PH), operation recording (OR), treatment options (TO), physical examination (PE), food, drug, and department. The source and specific definition of liver cancer entities are shown in Table 1.

**Table 1:** Eleven types of conceptual layer.

Entity type	Source	Definition
Patient	EMRs	Patient ID and status (such as age >40)
Examination	EMRs	CT, MRI
Symptom	EMRs and xywy.com	Left upper abdominal pain, vomiting
Diseases	EMRs and xywy.com	Liver cancer
Past history	EMRs	Smoking history
Operation recording	EMRs	Cholecystectomy
Treatment options	EMRs	Laparoscopic right hepatic cancer resection
Physical examination	EMRs	Abdominal distension
Food	xywy.com	Egg
Drug	xywy.com	Luolian Jiaonang
Department	xywy.com	Surgical oncology

**Figure 2:**
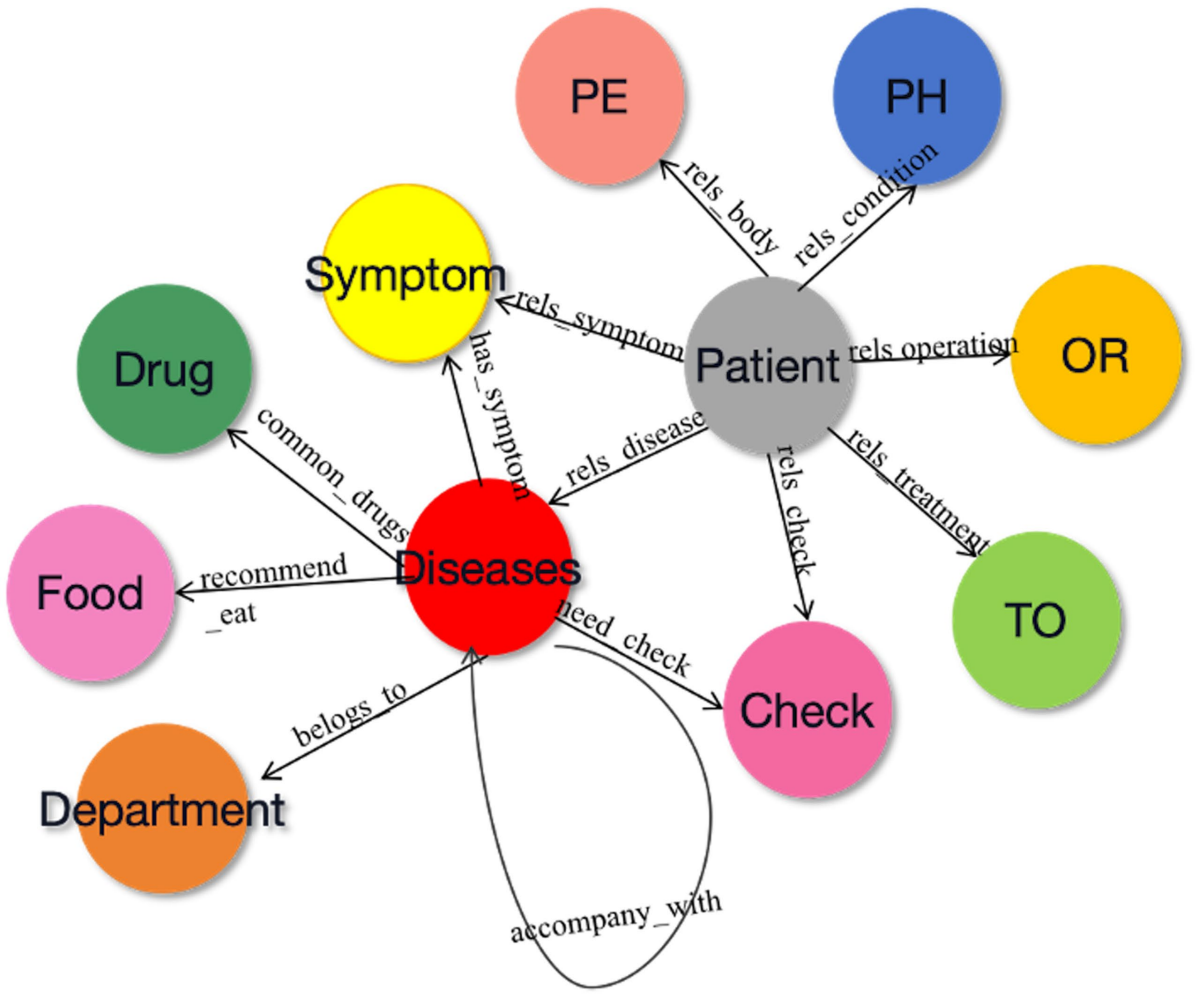
The relationship between different conceptual layers.

In accordance with the three-element principle of KG construction, it is essential to define three core components during the design of the conceptual layer: subject entity, relationship, and object entity. With the help of hospital experts, 11 relationships between entities and attributes are defined in this study, as shown in Figure 2. The starting node of the arrow is the subject entity pointing to the object entity, and the content on the arrow is the relationship. For example, within the triad <disease-has_symptom-symptom>, “disease” serves as the subject entity, “symptom” serves as the object entity, and “has_symptom” denotes the relationship between the subject and object.

### 3.3 Data preprocessing

The chosen EMRs underwent a de-identification process to ensure patient privacy. Specifically, all personally identifiable information, including patient names, ID numbers, addresses, and contact details, was removed or replaced with randomly generated patient IDs. Each EMR was assigned a unique code to preserve data traceability during annotation while preventing re-identification.

In addition, normalization was applied to standardize the clinical text and improve annotation consistency. This included unifying date and time formats, standardizing measurement units and laboratory values, correcting typographical errors and removing redundant symbols or formatting inconsistencies.

**Figure 3:**
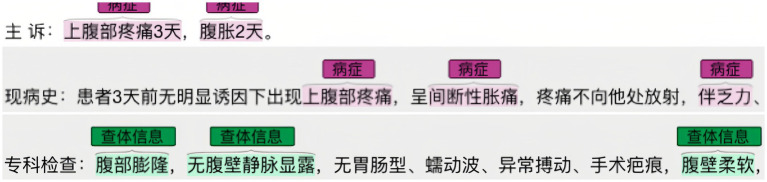
Example of a real-world liver cancer Chinese EMR annotation.

**Figure 4:**
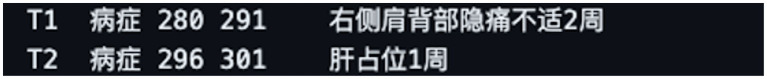
Ann-Brat format annotation.

Once the conceptual layer was designed, real-world Chinese EMRs were annotated using Colabeler^2^. Figure 3 shows an example of annotation in the “disease” entity. The annotation results were saved in the Ann-Brat format, as shown in Figure 4. “T1” denotes the first entity in the text, disease is the entity type. Numbers 280 and 291 are the start and end positions of the disease entity in the text, respectively. The phrase ‘dull pain in the right shoulder and back for 2 weeks’ is the disease name of the entity. Finally, the annotated documents were converted to the Ann-Brat format.

For the semi-structured data on the XYWY website, we crawled information such as disease common knowledge, diagnostic methods, and treatment plans. Through the hierarchy of paragraphs, titles, and hyperlink information of subtitles, attributes of conditions can be identified and extracted.

### 3.4 Named entity recognition

**Figure 5:**
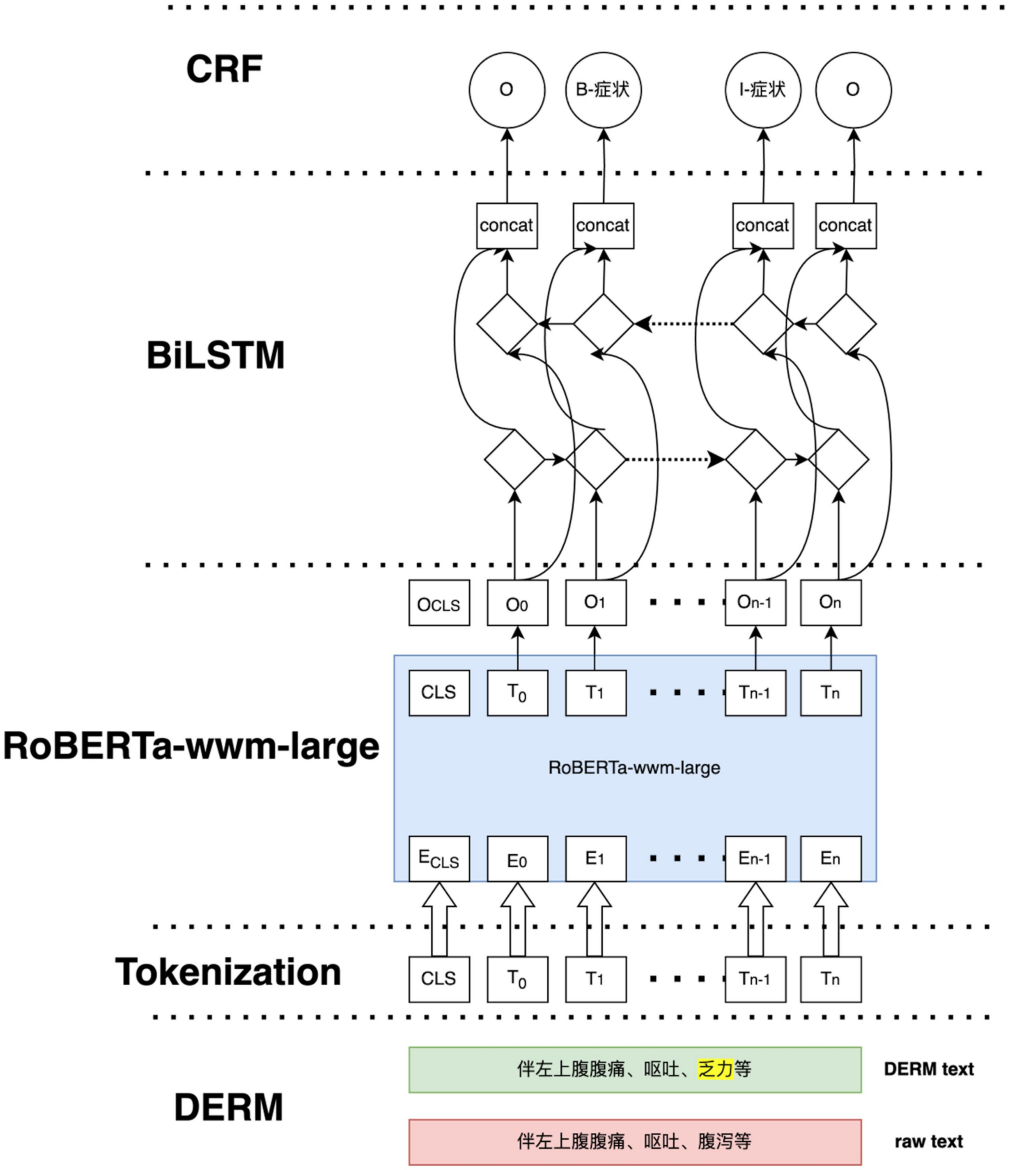
The framework of DERM-RoBERTa-wwm-large-BiLSTM-CRF.

In this study, the DERM-RoBERTa-wwm-large-BiLSTM-CRF deep learning model for liver cancer entity recognition was introduced. The overall structure of this model is shown in Figure 5. First, the DERM module replaces medical entities of the input sequence with standardized terms or masks certain parts of the text. Then, the processed text is fed into the RoBERTa-wwm-large model to obtain high-dimension vector representations. Next, the vector representations are fed into the BiLSTM network to extract the contextual dependencies of the sequence. Finally, the output of the BiLSTM layer is combined and passed to the CRF layer for decoding to output label dependencies and ensure valid label sequences.

**Figure 6:**
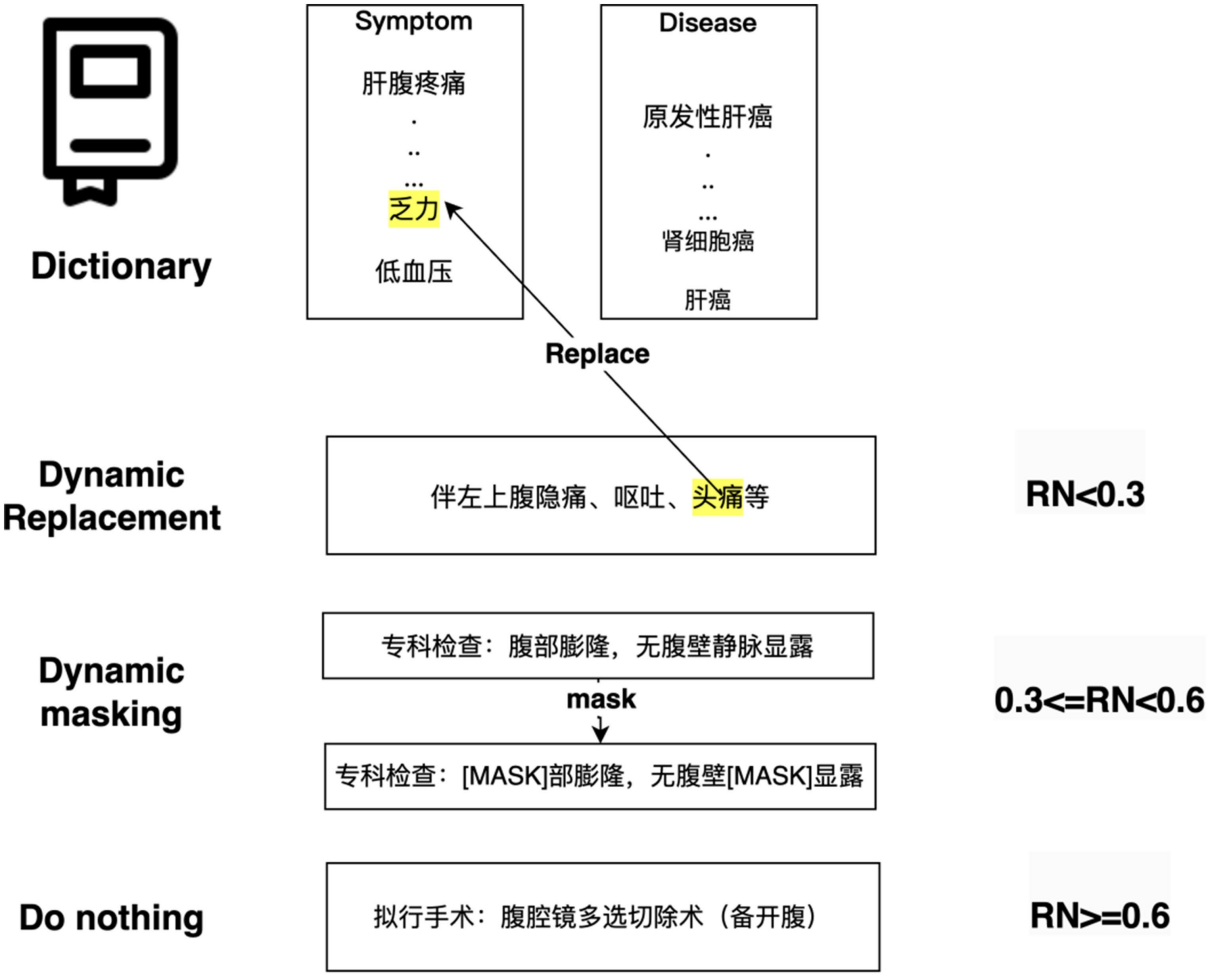
Hybrid data augmentation strategy for the training set.

DERM is a strategy used to process entities in NLP tasks, it helps to address the scarcity and imbalance of data. First, dictionaries from the Chinese Medical Entity Extraction dataset^3^ are constructed for different entities, including disease, symptom, treatment, and examination, and then dynamic entity replacement and masking are performed on the text of the EMRs during the training process. The replacement and masking strategies are shown in Figure 6. For all sequences in the EMRs, a random number (0 < RN < 1) was used to determine whether the entity in the sequence was replaced, masked, or did nothing. If RN < 0.3, the entities in the constructed dictionary are selected to replace those in the sequence. If 0.3 ≤ RN < 0.6, a masking strategy is used. If RN ≥ 0.6, the entities in the sequence remain the same.

#### 3.4.1 RoBERTa-wwm-large module

The RoBERTa-wwm-large model was selected for this study due to its distinct advantages over other BERT-based variants, particularly for processing Chinese text. The choice was guided by two primary factors. First, its implementation of Whole Word Masking (WWM) is critical for the Chinese language. Unlike standard masking that operates on individual characters, WWM masks entire words, which is better suited for capturing the holistic semantics of Chinese words that often comprise multiple characters, thereby mitigating potential word segmentation ambiguities. Second, RoBERTa features a more robust pre-training methodology. It optimizes the original BERT architecture by training on a larger corpus, using dynamic masking, and removing the next-sentence prediction (NSP) objective. These enhancements lead to more powerful and nuanced contextual embedding, which is especially beneficial for specialized domains. RoBERTa-wwm-large leverages extensive pre-training on a large-scale corpus of textual data. This -pre-training enables the model to capture the contextual representations of the input sequences. In this study, a 24-layer RoBERTa-wwm-large model is used, which is a stack of 24 encoders.

First, the input sequences are tokenized into subworlds using Byte-Pair Encoding (BPE), with special tokens [CLS] and [SEP] incorporated to denote the beginning and end of each sequence. Each token is mapped to a high-dimensional embedding of three features of tokens, which includes token embedding ( $E_{t}$ ), position embedding ( $E_{p}$ ), and segment embedding ( $E_{s}$ ). The resulting input to the RoBERTa-wwm-large model can be obtained by Equation 1:


(1) $$E_{input}=E_{t}+E_{p}+E_{s}$$

The $E_{\mathrm{input}}$ passes through multiple Transformer encoder layers, and each layer updates the token representations using a self-attention mechanism, as shown in Equation 2:


(2) $$Attention\left(Q,K,V\right)=\mathrm{Softmax}\left(\frac{QK^{T}}{\sqrt{dk}}\right)$$

where *Q, K,* and *V* are the word vector matrices derived from $E_{\mathrm{input}}$ . dk is the dimension of embedding. To capture diverse semantic relationships, multiple parallel self-attention heads were employed, as shown in Equation 3:


(3) $$\begin{array}{l} MultidHead\left(Q,K,V\right)\\ =Concat\left(head_{1},head_{2},\ldots head_{i}\ldots head_{n}\right)W_{o} \end{array}$$

where each head represents projections of Q , K , and V . $W_{o}$ is the token embedding. Finally, the output of RoBERTa-wwm-large is a sequence of embedding, one for each token in the input, as shown in Equation 4:


(4) $$H=H_{CLS},H_{1},H_{2},\ldots ,H_{n},H_{SEP}$$

where $H_{i}\in R^{d}$ , d is the dimension of the embedding space. These embeddings contain rich contextual information and serve as input for the subsequent BiLSTM layer.

#### 3.4.2 BiLSTM module

The BiLSTM module captures long-term dependencies and contextual information from both the forward and backward directions of the input embedding. Each embedding H_i_ is fed into the BiLSTM as an input vector at the i_th_ time step. The forward LSTM processes the embedding $\left[H_{1}{\mathrm{,H}}_{2},\ldots {\mathrm{,H}}_{i},\ldots H_{n}\right]$ to obtain the sequence of the forward hidden states ${\vec{h}}_{i}$ . The backward LSTM processes the embeddings to obtain $\left[H_{n}{\mathrm{,H}}_{n-1},\ldots {\mathrm{,H}}_{i},\ldots {\mathrm{,H}}_{2}{\mathrm{,H}}_{1}\right]$ , which is the sequence of the backward hidden state ${\overset{\leftarrow }{h}}_{i}$ .

The two LSTMs operate independently but simultaneously to capture both past and future dependencies in the sequence. At each time step *i*, the forward hidden state ${\vec{h}}_{i}$ and backward hidden state ${\overset{\leftarrow }{h}}_{i}$ are concatenated to form a combined representation: $h_{i}=\mathrm{Concat}\left({\vec{h}}_{i}{,\overset{\leftarrow }{h}}_{i}\right)$ , where $h_{i}\in R^{2h}$ , and h is the dimension of the hidden state in each LSTM. This concatenation ensures that each token representation at every time step incorporates both the preceding and succeeding contexts. On the sequence of scores $\left[s_{1}{\mathrm{,s}}_{2}\ldots {\mathrm{,s}}_{n}\right]$ calculated by BiLSTM hidden states as shown in Equation 5.


(5) $$S_{i}=Wh_{i}+b$$

where $S_{i}\in R^{k}$ is the score vector for k possible labels, and $W\in R^{k*2h}$ and $b\in R^{k}$ are the trainable weights and biases.

#### 3.4.3 CRF module

The CRF module plays a crucial role in NER. Instead of making independent predictions for each token, CRF jointly models the relationships across the entire sequence to ensure that the predicted labels are consistent with one another. In this study, the CRF layer operates a score was assigned to the labels of the input sequence, as shown in Equation 6:


(6) $$Score\left(X,y\right)=\overset{n}{\underset{i=1}{\sum }}S_{i,y_{i}}+\overset{n-1}{\underset{i=1}{\sum }}T_{y_{i},y_{i+1}}$$

where X represents the input text sequence and y represents the sequence of labels. $S_{i,y_{i}}$ is the score of the ith label of the ith word, $T_{y_{i},y_{i+1}}$ denotes the score when label $y_{i}$ turns into label $y_{i+1}$ . The probability of the prediction sequence is computed using Equation 7:


(7) $$p\left(y|X\right)=\frac{\exp\left(Score\left(X,y\right)\right)}{\sum_{y\prime }\exp\left(Score\left(X,y^{\prime }\right)\right)}$$

As a result, it computes the relative probability of a specific sequence y compared to all other possible label sequences for the input X . Finally, at inference time, the goal is to find the label sequence $y^{*}$ with the highest score, as shown in Equation 8:


(8) $$y^{*}=argmaxScore\left(X,y^{\prime }\right)$$

### 3.5 Knowledge fusion

The KF addresses data redundancy, inconsistency, and incompleteness in KG construction, thereby enhancing the quality and utility of the resulting KG. KF performed after NER is applied specifically to RLC-EMRs. This ensured the data are integrated accurately and consistently.

In real-world Chinese EMRs, patient medical records are generally written by different doctors. Because different doctors have different recording habits and terminology, some entity names in EMRs are inconsistent. In addition, there are some inconsistencies between the entity names in EMRs and those of the professional website XYWY.com. Therefore, the extracted entities are different. For example, while EMRs often use Primary Hepatocellular Carcinoma(原发性肝细胞癌), healthcare websites such as XYWY.com use the simplified term Hepatocellular Carcinoma (原发性肝癌). In other cases, the standardized surgical term Laparoscopic Liver Tumor Excision (腹腔镜肝肿瘤切除术) also demonstrates term variation in clinical practice. Some doctors document this term as Laparoscopic Liver Cancer Resection (腹腔镜肝癌切除), while others record it as Laparoscopic Liver Tumor Resection (腹腔镜肝肿物切除术) in their EMR. Although both diseases and operations refer to the same entity, the difference in terminology results in the appearance of two different entities. This discrepancy can cause problems in entity recognition, information extraction, and KG construction. Therefore, a KF is required to map different entities to a standard entity.

This study utilizes Term Frequency-Inverse Document Frequency (TF-IDF) for the KF, a statistical technique commonly applied in text mining and information retrieval to measure the relevance of entities within a corpus. The TF-IDF approach is particularly useful in the task of KF, where the goal is to identify and normalize entities that may be expressed differently in various sources, such as medical records and professional websites.

TF-IDF consists of two key elements: Term Frequency (TF), which captures how often an entity appears in a document, and Inverse Document Frequency (IDF). The formula for the TF is outlined in Equation 9 as follows:


(9) $$TF\left(t,d\right)=\frac{f_{t,d}}{f_{d}}$$

where $f_{t,d}$ represents the number of occurrences of entity t in document d , and dd represents the total number of terms in document d . The higher the TF(t,d) value, the more important the entity t is within that document.

The IDF measures the distinctiveness of an entity across the entire corpus. The idea behind IDF is that common entities that appear in many documents should be weighted less, as they do not provide as much information in Equation 10:


(10) $$IDF\left(t\right)=\log\left(\frac{N}{\left|d\in D:t\in d\right|}\right)$$

where N represents the total number of documents in the corpus, and |d∈D:t∈d| represents the number of documents that contain an entity t . IDF assigns a higher weight to entities that are rare across the corpus, making them more distinctive.

Then, the TF-IDF value for an entity t in a document d is obtained by multiplying the TF and IDF values, as in Equation 11:


(11) TF−IDF(t,d)=TF(t,d)∗IDF(t)

Finally, to normalize entities from the EMRs to the standard entities found in XYWY.com and CCMT-2019, we computed the cosine similarity between the TF-IDF vectors of entities in the input $v_{1}$ and entity $v_{2}$ from the reference corpus. The cosine similarity between two TF-IDF vectors $v_{1}$ and $v_{2}$ is calculated using Equation 12:


(12) $$\mathrm{consine similarity}\left(V_{1}{\mathrm{,V}}_{2}\right)=\frac{V_{1}\cdot V_{2}}{V_{1}V_{2}}$$

This measures the angle between the vectors, where a cosine similarity closer to 1 indicates a high similarity between the two entities. For disease and symptom entities, XYWY.com serves as a reference corpus for normalization, aligning entities with EMRs. For other clinical entities, such as treatments and operation recording. CCMT-2019 as a reference corpus only normalizes these entities. This combined approach enables the seamless integration of medical data from disparate sources.

To systematically implement this knowledge fusion process, we propose Algorithm 1, which integrates the TF-IDF similarity calculation with threshold-based decision making for automated and manual entity mapping.

**ALGORITHM 1:**
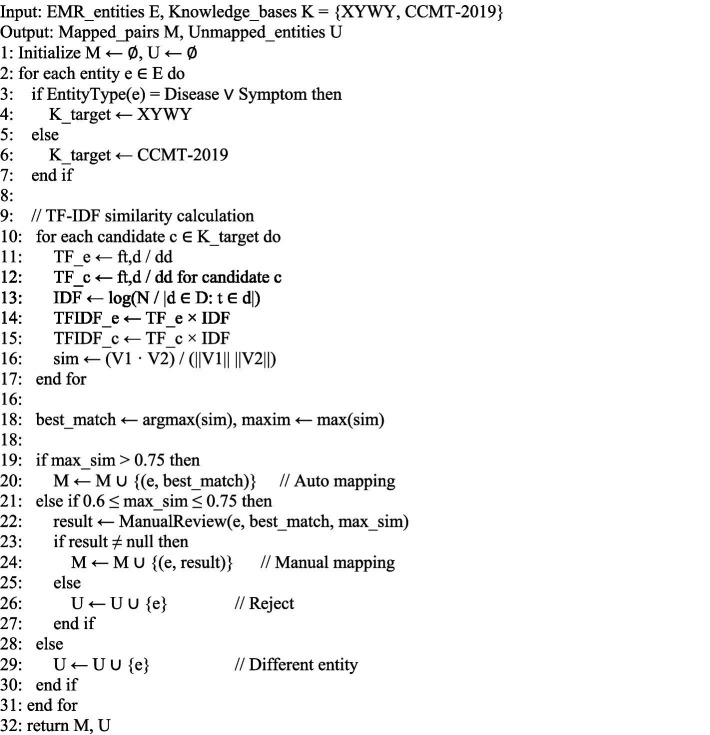
Knowledge fusion for EMR entity mapping.

Algorithm 1 demonstrates the complete workflow for entity normalization, where entities are first classified by type, then similarity scores are computed using the TF-IDF approach described in Equations 9–12, and finally mapped based on predefined similarity thresholds.

This approach ensures high-confidence automatic mappings (similarity > 0.75) while allowing manual review for borderline cases (0.6 ≤ similarity ≤ 0.75). Entities with similarity scores below 0.6 are considered as different entities that cannot be reliably mapped to existing knowledge bases. The algorithm employs a three-tier mapping strategy: (1) Automatic mapping for high-confidence matches ensures efficiency in processing clearly related entities; (2) Manual review for moderate-confidence matches maintains accuracy by incorporating human expertise for ambiguous cases; (3) Rejection for low-confidence matches prevents false mappings that could introduce noise into the knowledge graph. This balanced approach optimizes both precision and recall in the entity normalization process while maintaining computational efficiency.

### 3.6 Knowledge graph construction, visualization and quality assessment

This study utilized the Neo4j graph database to construct the liver cancer KG. Unlike traditional relational databases, a graph database is designed to represent and store ontologically structured knowledge, thereby enabling the visualization of complex relationships between entities. Neo4j supports ACID-compliant transactions, ensures data integrity, and uses Cypher, a query language designed for querying graph data, which is both simple in syntax and efficient, regardless of the size of dataset. For this study, Neo4j was chosen to manage and visualize the data in the Liver Cancer KG. To enhance the usability and readability of the graph, the KG displays the top three-tier structure of the liver cancer KG by default. Users can navigate through the graph using the Neo4j node expansion feature to explore the different layers of information. To enhance the visual clarity of the graph, nodes at different levels were distinguished by color. For instance, “Disease” nodes are represented in yellow, while “Symptom” nodes are colored green. The treatment nodes are also depicted in green. In addition, the semantic relationships between entities are differentiated using specific color entities. The Liver Cancer KG constructed in this study includes entities 46,364 and 296,655 semantic relationships, covering a wide range of liver cancer-related topics such as symptoms, treatment options and physical examination.

The factual quality of the KG was quantified using Triple Accuracy. This metric is defined as the proportion of clinically correct triples among all sampled triples deemed evaluable. S denote a set of sampled triples. For each triple i∈S , an expert annotator assigned a label $y_{i}\in \left\{Correct\mathrm{,Incorrect,Insufficient Context}\;\left(\mathrm{IC}\right)\right\}$ . The number of evaluable triples ( $n_{eff}$ ) and the number of correct triples ( x ) were defined as Equation 13:


(13) $$n_{eff}=\underset{i\in S}{\sum }1\left\{y_{i}\in \left\{Correct,Incorrect\right\}\right\},x=\underset{i\in S}{\sum }1\left\{y_{i}=Correct\right\}$$

Where 1{�} is the indicator function. The Triple Accuracy (TAcc) was subsequently calculated as:


(14) $$\mathrm{TAcc}=\frac{x}{n_{eff}}$$

Triples labeled as IC were excluded from this calculation, and their proportion was reported separately as an indicator of documentation completeness or the need for alignment refinement. A stratified random sample of 500 triples was drawn from the final, de-identified KG. The stratification was based on major relation types include ‘has_symptom’, ‘accompany_with’, ‘recommends_drug’ ‘recommends_eat’ and ‘rels_diease’.

## 4 Results

### 4.1 Evaluation metrics

To quantitatively assess the performance of the NER model, these metrics are defined in Equations 13–17. The True Positives (TP) denote the number of predicted entities that exactly match the ground truth in both type and boundary, False Positives (FP) represent predicted entities that are not present in the ground truth (including cases with incorrect type or boundary), and False Negatives (FN) refer to ground-truth entities that were not detected by the model. Precision measures the proportion of correctly identified entities among all predicted entities. It indicates the model’s accuracy in positive predictions. Recall measures the proportion of actual entities that the model correctly identifies. It reflects the model’s ability to capture all relevant instances. The F1 score is the harmonic mean of P and R. It provides a balanced evaluation metric that accounts for both FP and FN.


(15) $$P=\frac{TP}{TP+FP}$$

(16) $$R=\frac{TP}{TP+FN}$$

(17) $$F1=\frac{2*\left(P*R\right)}{\left(P+R\right)}$$

### 4.2 CMeEE-v2 experimental results and analysis

The experimental results on the CMeEE-v2 dataset demonstrated the performance of the different models through comprehensive analysis. Table 2 presents the precision recall and F1 scores of multiple models, including the proposed DERM-RoBERTa-wwm-large-BiLSTM-CRF model, compared with several baseline models.

**Table 2:** Comparison of the proposed method with the prior works on CMeEE-v2.

Model	Precision	Recall	F1
Lattice-LSTM (Zhang and Yang, 2018)	61.26%	62.33%	61.79%
Simple-Lexicon (Ma et al., 2019)	61.00%	60.31%	60.64%
FLAT (Li et al., 2020a; Li et al., 2020b; Li L. et al., 2020)	61.83%	66.42%	64.03%
TPORE (Emami et al., 2020)	63.73%	66.25%	64.94%
ChatGLM-6B (Xu et al., 2024)	–	–	67.45%
GPT-4 (Yang et al., 2024)	–	–	65.42%
DERM-RoBERTa-wwm-large-BiLSTM-CRF (Ours)	68.50%	69.18%	68.84%

The comparison reveals that the DERM-RoBERTa-wwm-large-BiLSTM-CRF model significantly outperforms existing baseline models on the CMeEE-v2 dataset. The DERM-RoBERTa-wwm-large-BiLSTM-CRF model achieved an F1 score of 68.84%. This represents a substantial improvement over TPORE at 64.94% and FLAT at 64.03%. The recall performance of DERM-RoBERTa-wwm-large-BiLSTM-CRF reaches 69.18% which is comparable to FLAT at 66.42%. The model demonstrated exceptional precision at 68.50% and surpassed all the other models. Additionally, our model exceeds the performance of recent LLM-based approaches, including ChatGLM-6B with advanced decoding strategies at 67.45% F1 score, GPT-4 under few-shot prompting at 57.2% F1 score, and ChatGPT GPT-3.5 under few-shot prompting at 46.9% F1 score. These results indicate that DERM-RoBERTa-wwm-large-BiLSTM-CRF possesses clear advantages for entity recognition tasks, showing significant performance improvements compared to baseline models such as Simple-Lexicon and Lattice-LSTMTo explore the contribution of each module within the DERM-RoBERTa-wwm-large-BiLSTM-CRF model, a series of ablation experiments were conducted. M1 represents DERM, and M2 represents the BiLSTM-CRF module. The experiments gradually removed different modules and compared the changes in accuracy, recall, and F1 score. The results are presented in Table 3.

**Table 3:** Ablation study of the model on CMeEE-v2.

Experiment number	With		Precision	Recall	F1
	M1	M2			
Experiment 1	×	×	63.12%	60.22%	61.65%
Experiment 2	×	√	62.62%	68.00%	65.20%
Experiment 3	√	×	68.39%	67.85%	68.13%
Experiment 4	√	√	68.50%	69.18%	68.84%

In Experiment 1, both M1 and M2 were removed, leaving the base RoBERTa-wwm-large model. The accuracy was 63.12%, the recall was 60.22%, and the F1 score was 61.65%. In Experiment 2, only the BiLSTM-CRF module was used. The accuracy was 62.62%, recall increased to 68.00%, and F1 score rose to 65.20%. In Experiment 3, only the DERM module was used. The accuracy was 68.39%, the recall was 67.85%, and the F1 score was 68.13%. In Experiment 4, all modules were kept, which was the full model structure. The accuracy reached 68.50%, the recall was 69.18%, and F1 score was 68.84%. This was the best performance among all experiments.

The results show that adding any module improves model performance. Compared to the baseline model, maintaining the BiLSTM-CRF (Experiment 2) increased recall and F1 score. This shows that BiLSTM-CRF is important for optimizing label dependencies and improving entity coverage. The DERM strategy (Experiment 3) led to a greater performance improvement. Its F1 score was close to the full model, indicating that DERM enhances semantic modeling and contextual understanding. Finally, the full model (Experiment 4) performed best in all three metrics. This shows that the integration of M1 and M2 modules has complementary advantages. The DERM strategy enhances the generalization ability of model for complex entities. The BiLSTM-CRF structure improves the modeling of label sequence dependencies.

### 4.3 RLC-EMRs experimental results and analysis

#### 4.3.1 Entity recognition results and analysis

In this study, entity recognition was performed based on the definitions provided in the conceptual layer. Table 4 presents a comprehensive comparison of the entity counts before and after the fusion process. The initial entity recognition identified 11 distinct entity types with significant variations in their quantities. Notably, after the fusion process, certain entity categories, such as Examination, Diseases, and Symptoms, showed substantial increases in their numbers. For example, the number of disease entities increased from 449 to 9,037, while symptom entities expanded from 136 to 6,789. Additionally, new entity types emerged post-fusion, including food (4,870), drug (3,828), and department (54). Table 5 provides detailed statistics on the relationships between different entity types in the KG. The relationship distribution reveals that “recommand_drug” and “has_symptom” are the most frequent relationships, with 59,467 and 54,717 instances, respectively. Food recommendations also played a significant role, with “recommand_eat” (40,236), “no_eat” (22,247), and “do_eat” (22,238) relationships being prominent. Clinical relationships, such as “rels_diseases” (15,289) and “acompany_with” (12,029), demonstrate the complex interconnections between different medical entities in the KG.

**Table 4:** Statistics on the number of entities.

Entity type	Number (before fusion)	Number (after fusion)
Patients	304	304
Examination	113	3,677
Diseases	449	9,037
Symptom	136	6,789
Past history	420	420
Operation recording	337	337
Treatment options	420	420
Physical examination	324	325
Food	/	4,870
Drug	/	3,828
Department	/	54

**Table 5:** Statistics on the relationship of entities.

Relationship type	Number
recommand_drug	59,467
has_symptom	54,717
recommand_eat	40,236
need_check	39,423
no_eat	22,247
do_eat	22,238
drugs_of	17,315
rels_diseases	15,289
common_drug	14,649
acompany_with	12,029
belongs_to	8,844
rels_body	1,536
rels_operation	752
rels_disease	695
rels_symptom	584
rels_check	377
rels_treatment	288
rels_condition	240

A Python script was then employed to transform the Ann-Brat format to the BIO format, which is often used as the standard format for NER tasks. The dataset was split into training, validation, and testing subsets in an 8:1:1 ratio and subsequently fed into the deep learning model for processing. We conducted experiments to evaluate and compare the performance of the four models in recognizing entities from EMRs. The models include DERM-RoBERTa-wwm-large-BiLSTM-CRF, RoBERTa-wwm-large-BiLSTM-CRF (Cui et al., 2023), DERM-BERT-large-BiLSTM-CRF, BERT-large-BiLSTM-CRF (Dai et al., 2019), GPT-4, ChatGLM-6B and Word2vec-BiLSTM-CRF (Luo et al., 2018). The experiments focus on seven types of entities in EMRs: Examination, Disease, Symptom, Past History, Operating Recording, Treatment Options, and Physical Examination the results of the four models are shown in Table 6.

**Table 6:** Entity recognition evaluation result of different models in the EMRs.

Model	Precision	Recall	F1Score
DERM-RoBERTa-wwm-large-BiLSTM-CRF (Ours)	95.36%	93.94%	94.65%
RoBERTa-wwm-large-BiLSTM-CRF	94.46%	92.23%	93.84%
DERM-BERT-large-BiLSTM-CRF	94.69%	93.24%	93.96%
BERT-large-BiLSTM-CRF	89.69%	90.98%	90.33%
GPT-4	88.42%	86.75%	87.58%
ChatGLM-6B	82.13%	79.86%	80.98%
Word2vec-BiLSTM-CRF	69.29%	67.53%	68.4%

The proposed DERM-RoBERTa-wwm-large-BiLSTM-CRF model (F1 score: 94.65%, precision: 95.36%, recall: 93.94%) demonstrates substantial improvements over the BERT-large-BiLSTM-CRF baseline model (F1 score: 90.33%, precision: 89.69%, recall: 90.98%). Specifically, the model achieves improvements of 4.3% in F1 score, 5.8% in precision, and 3.0% in recall compared to the baseline. These significant improvements indicate the effectiveness of domain-specific adaptations and advanced pre-training strategies.

The Word2vec-BiLSTM-CRF model (F1 score: 68.40%, precision: 69.29%, recall: 67.53%) showed considerably lower performance compared to the DERM-BERT-large-BiLSTM-CRF model (F1 score: 93.96%, precision: 94.69%, recall: 93.24%), with differences of 25.97, 25.40, and 25.14% in precision, recall, and F1 score, respectively. This demonstrates the superiority of contextualized word embedding over static embedding in EMR entity recognition.

The RoBERTa-wwm-large-BiLSTM-CRF model (F1 score: 93.84%, precision: 94.46%, recall: 93.23%) also significantly outperformed the Word2vec-BiLSTM-CRF baseline, showing improvements of 25.04, 25.17, and 25.00% in precision, recall, and F1 score, respectively. This highlights the advantages of advanced pre-training strategies in capturing contextualized features.

The DERM-RoBERTa-wwm-large-BiLSTM-CRF model demonstrated consistent improvements over RoBERTa-large-BiLSTM-CRF (F1 score: 93.84%, precision: 94.46%, recall: 93.23%), with increases of 0.80, 0.90, and 0.70% in accuracy, recall, and F1 score. Similarly, when compared to DERM-BERT-large-BiLSTM-CRF (F1 score: 93.96%, precision: 94.69%, recall: 93.24%), the model showed improvements of 0.68, 0.73, and 0.70% across these metrics.

The GPT-4 model (F1 score: 87.58%, precision: 88.42%, recall: 86.75%) showed considerable performance gaps compared to our proposed model, with differences of 7.07, 6.94, and 7.19% in F1 score, precision, and recall, respectively. This demonstrates the limitations of general-purpose large language models in specialized medical entity recognition tasks, even when employing few-shot learning strategies.

The ChatGLM-6B model (F1 score: 80.98%, precision: 82.13%, recall: 79.86%) exhibited even larger performance gaps compared to the proposed model, with differences of 13.67, 13.23, and 14.08% in F1 score, precision, and recall, respectively. These substantial differences highlight the advantages of domain-specific pre-training and task-specific architectural design over general-purpose language models in medical NER applications.

Table 7 shows the application of the DERM-RoBERTa-wwm-large-BiLSTM-CRF method to calculate F1 scores, precision, and recall for each of the seven significant entities within the test dataset. Analysis of the table reveals that the operation entity achieved the highest F1 score of 100%, while the symptoms entity recorded the lowest with an F1 score of 86.06%. This result demonstrates the model’s capacity for generalization in small sample datasets.

**Table 7:** Precision, recall, and F1 score in recognition of different entity types on DERM-RoBERTa-wwm-large-BiLSTM-CRF.

Entity type	Precision	Recall	F1
Disease	92.49%	93.02%	92.75%
Body check	91.59%	92.03%	91.80%
Symptom	85.57%	86.56%	86.06%
Condition	88.47%	88.98%	88.72%
Check	92.13%	92.13%	92.13%
Treatment	94.47%	93.86%	94.16%
Operation	100%	100%	100%

In conclusion, the DERM-Roberta-large-BiLSTM-CRF model achieved the best performance among all evaluated models, demonstrating that domain-specific fine-tuning combined with advanced pre-training strategies significantly enhances entity recognition in EMRs.

#### 4.3.2 Knowledge fusion results and analysis

**Figure 7:**
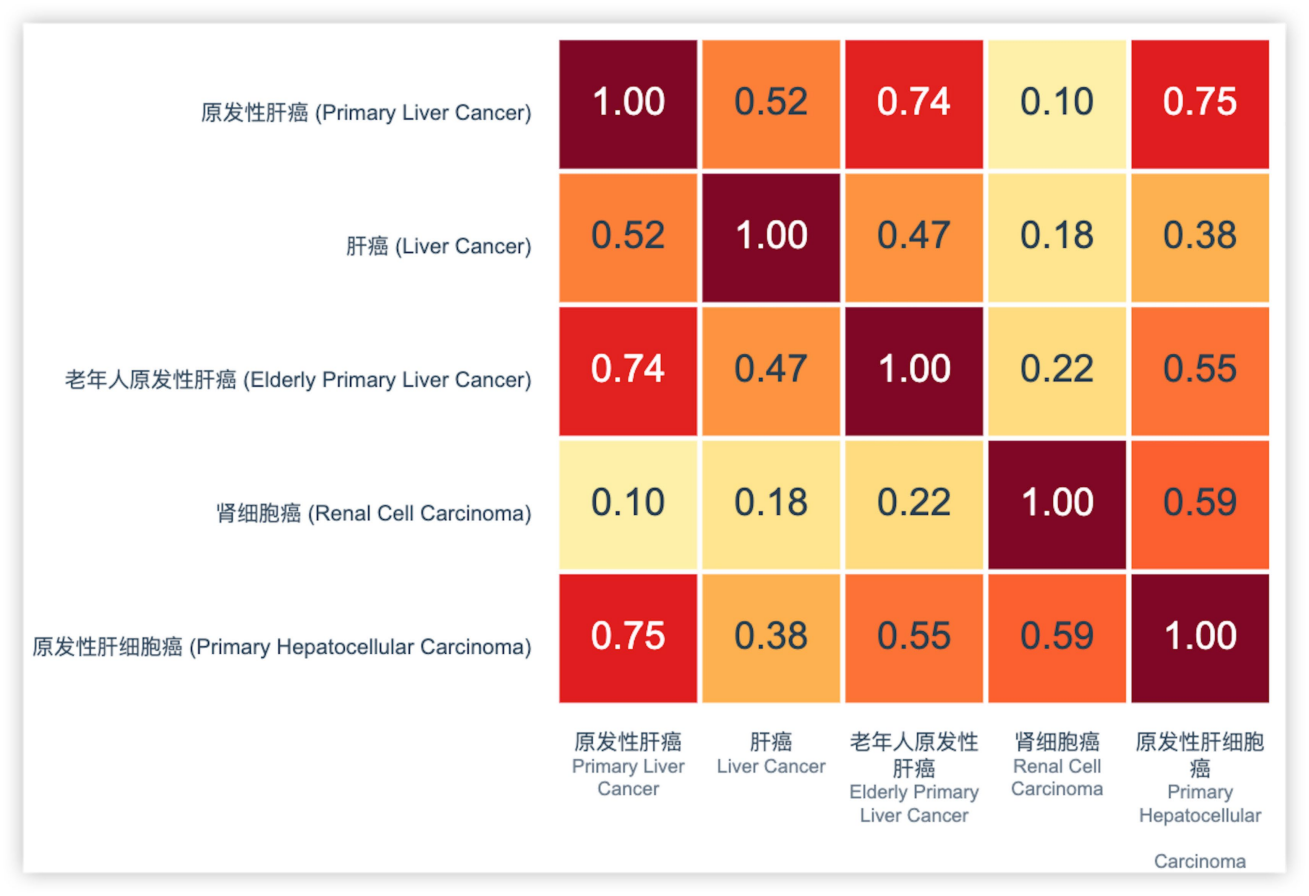
Visualization of TF-IDF vectors.

Figure 7 shows a heatmap of TF-IDF vector similarity scores among different liver cancer-related terms. The similarity matrix reveals significant semantic overlap between certain disease entities. Notably, hepatocellular carcinoma (原发性肝细胞癌) is in EMR, and the corpus content is primary peritoneal carcinoma (原发性肝癌), liver cancer (肝癌), renal cell carcinoma (肾细胞癌) and primary liver cancer of the elderly primary hepatocellular carcinoma (老年人原发性肝细胞癌). Hepatocellular carcinoma (原发性肝细胞癌) and primary peritoneal carcinoma (原发性肝癌) demonstrate a high cosine similarity score of 0.75, indicating substantial semantic equivalence. This strong correlation suggests these terms refer to the same clinical entity despite variations in terminology. Based on this high similarity score and medical domain knowledge, these entities were merged into our KG to maintain consistency and reduce redundancy. The fusion of these entities not only standardizes the disease representation but also enhances the overall quality and reliability of the KG structure.

**Figure 8:**
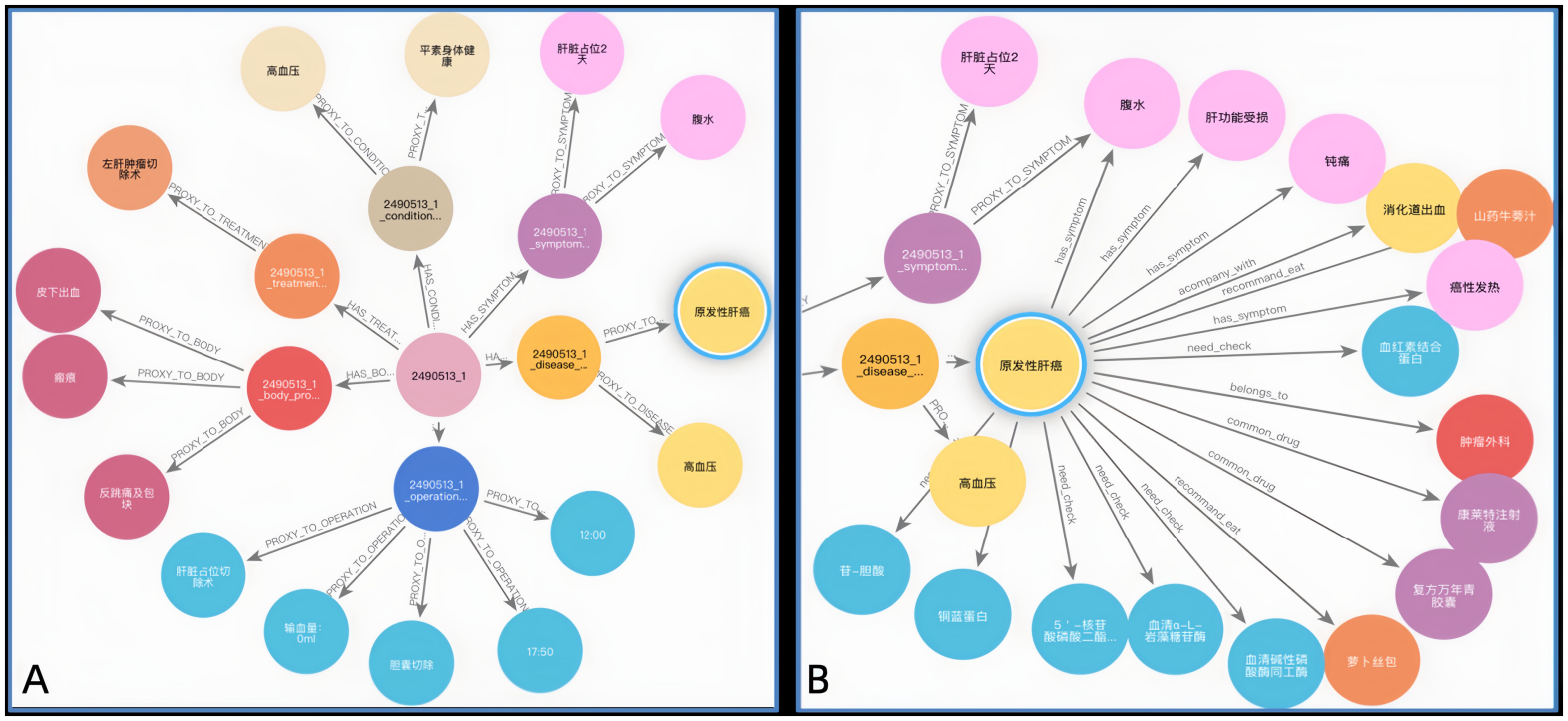
**(A)** KG before KF for Patient ID “2490513.” **(B)** KG after KF for Patient ID “2490513.”

Figures 8A,B illustrate the KG before and after KF for Patient ID “2,490,513_1.” This graph aligns disease and symptom entities with XYWY.com website, significantly enhancing the patients’ related entities and relationships. Such enrichment not only adds value to patient data but also facilitates future downstream applications of KG. First, the TF-IDF algorithm computes the vectorizer of the TF-IDF using the disease corpus of XYWY.com as input. Then, the disease entities appearing in the EMRs are used as queries, and the cosine similarity between each query and the entities in the TF-IDF vectorizer is calculated. Finally, a certain threshold is set, and the output with the highest similarity is used as the target matching entity for the query, thus completing the knowledge matching.

#### 4.3.3 KG construction results and analysis

We manually reviewed the knowledge graph to evaluate its factual accuracy. The overall triple accuracy was 93.5%. This high score confirms that the constructed KG is reliable. For this assessment, we sampled 500 triples from five major relation categories. Experts found that only 10 triples had IC and were excluded from the accuracy calculation. The remaining 490 triples were assessed for correctness against established clinical guidelines. Table 8 presents the detailed results. The analysis shows a consistently high accuracy across all relation types, which indicates our method for building the graph is robust. The ‘has_symptom’ relation had the highest accuracy at 94.3%. Other key relations were also very accurate. For example, ‘accompany_with’ scored 93.5%, ‘recommend_drug’ scored 93.2% and ‘recommend_eat’ were also highly reliable with an accuracy of 92.3%. The expert-validated accuracy across diverse topics confirms the factual integrity of the KG. Therefore, the graph provides a strong foundation for developing future tools, such as systems for clinical decision support or patient education.

**Table 8:** Triple accuracy by relation type.

Relation type	Sampled	Insufficient context	Denominator (=Sampled−IC)	Correct	Incorrect	Accuracy
has_symptom	160	2	158	149	9	94.30%
accompany_with	110	3	107	100	7	93.46%
recommand_drug	90	2	88	82	6	93.18%
recommand_eat	80	2	78	72	6	92.31%
rels_diseases	60	1	59	55	4	93.22%
Overall	500	10	490	458	32	93.47%

**Figure 9:**
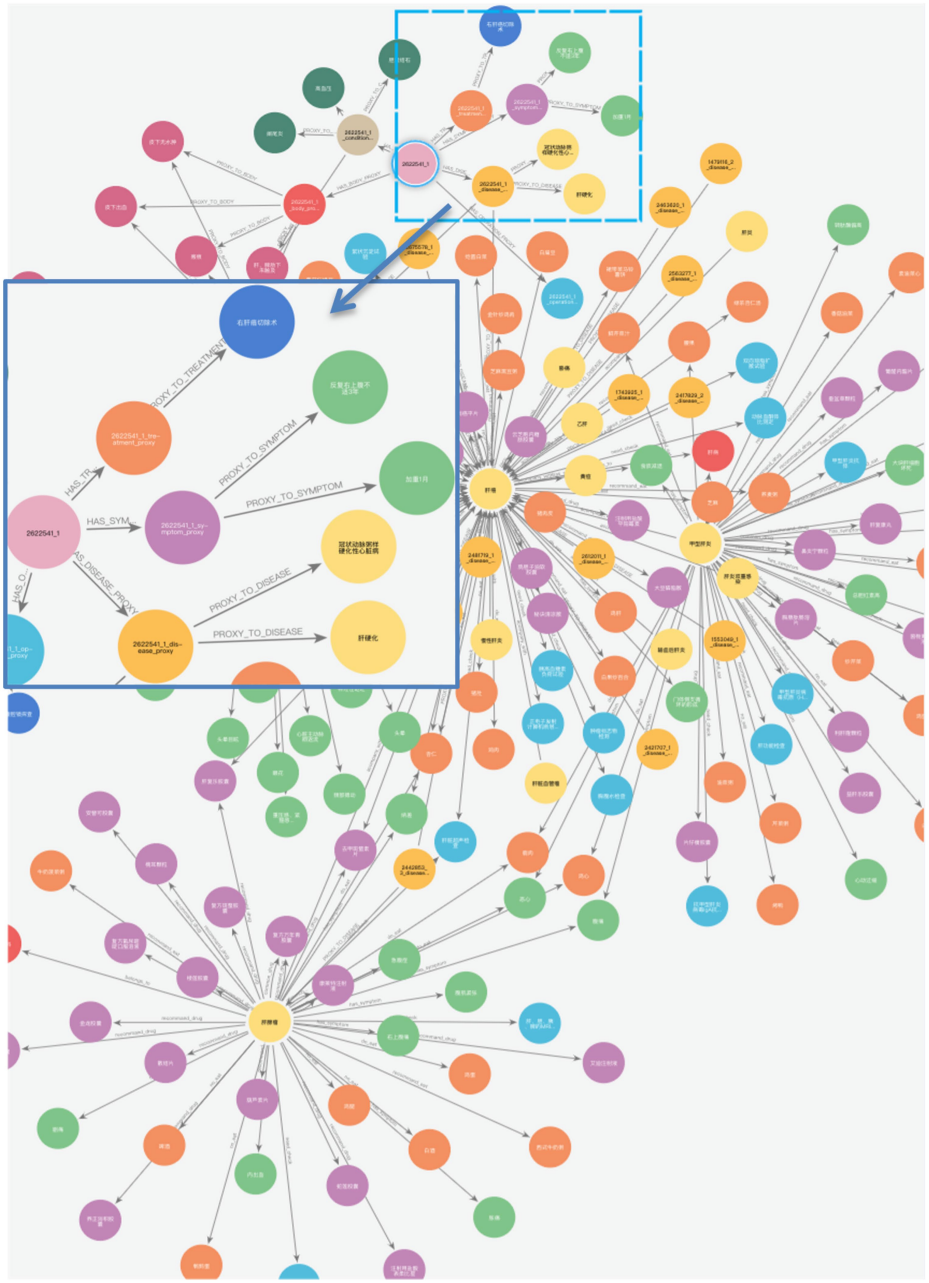
Overview of liver cancer KG.

**Figure 10:**
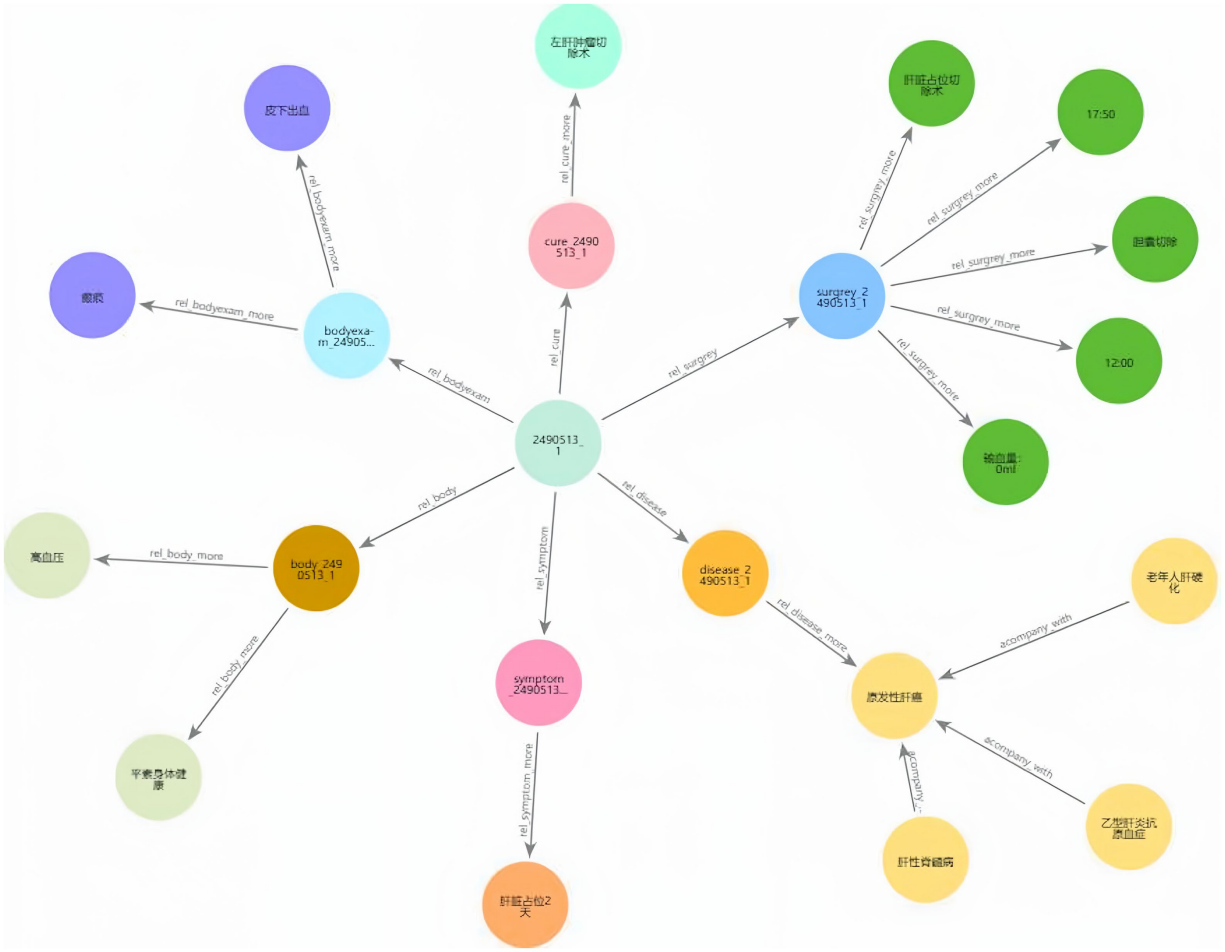
Patient ID “2490513_1” KG.

Finally, we constructed a comprehensive liver cancer KG containing 11 types of entities, with a total of 46,365 entities and 296,655 triples, as shown in Figure 9. The magnified section focuses on patient ID “2622541_1,” displaying the connections between the patient and their specific diseases, symptoms, and operation recording, demonstrating the practical application of our KG in representing individual patient cases. In addition, the patient entity has its basic attributes, such as nation, age, and sex. For example, Figure 10 shows that Patient 2,490,513_1 is used as the center to associate the proxy nodes, including examination, symptoms, diseases, past history, operation recording, treatment options, and physical examination. Then, based on these proxy nodes, specific disease, symptom, and treatment nodes are identified.

**Figure 11:**
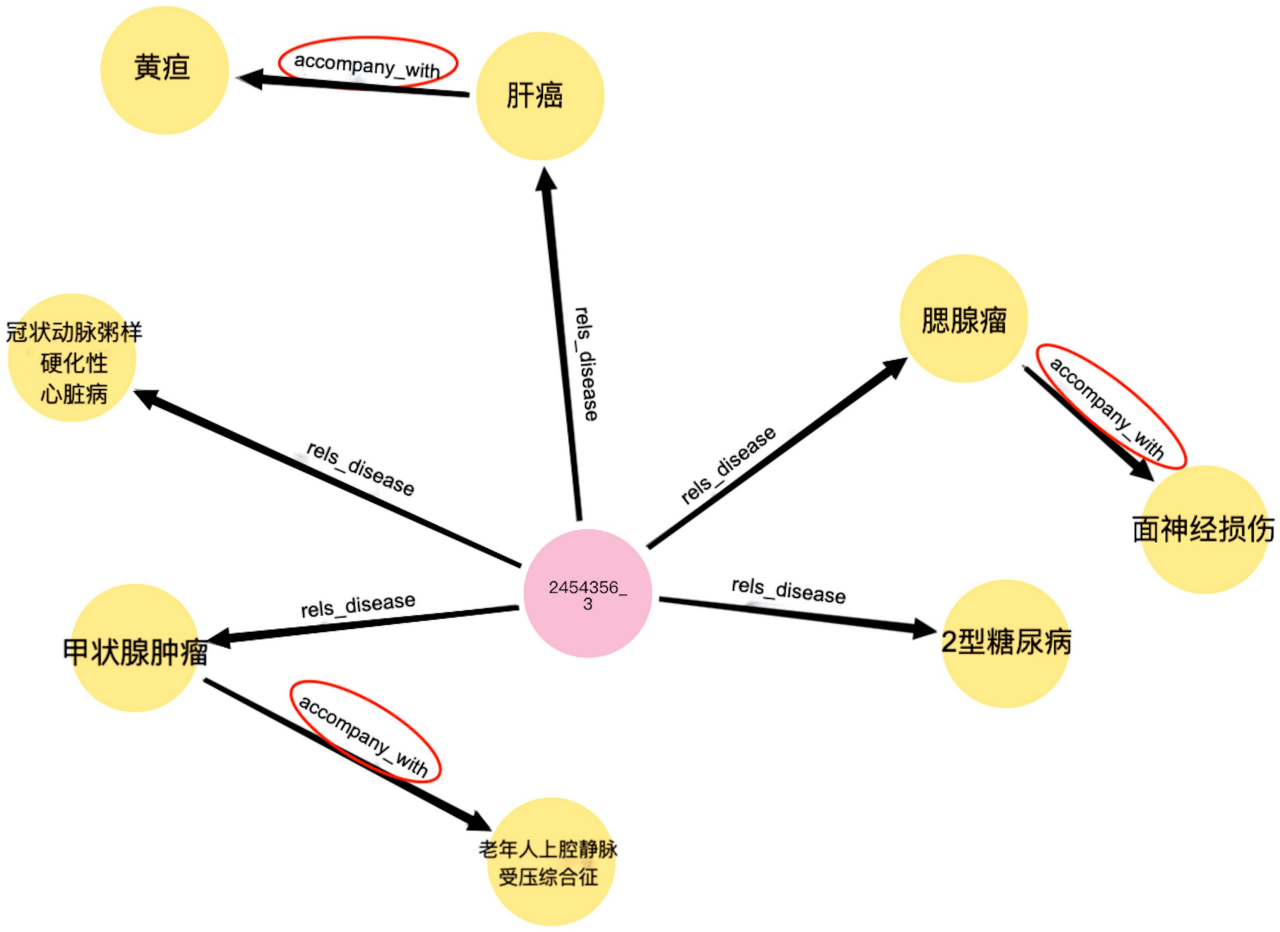
Sematic research result.

Complication retrieval is an application of liver cancer KGs, allowing the search and query of complications using keywords or logical relationships. Complication retrieval provides insights and references for medical professionals, helping to optimize diagnosis and treatment strategies. Utilizing KG for liver cancer, it can efficiently associate patients with diseases and diseases with complications in the form of a triple. Neo4j allows users to customize advanced Cypher queries. For example, the Cypher query statement can be used to query the diseases associated with patient ID “2454356_3” and their related complication s through the “rels_disease” and “accompany_with” relationships. As shown in Figure 11, it is quick and easy to identify a disease that is accompanied by complications.

## 5 Discussion

This study shows the feasibility and effectiveness of building a domain-specific liver cancer KG from diverse Chinese real-world data sources. We integrated EMRs, standardized medical terms, and reliable online medical resources. Our DERM-RoBERTa-wwm-large-BiLSTM-CRF model outperformed strong baseline models in NER. It achieved this on both the public CMeEE-v2 dataset and our private RLC-EMRs dataset. These gains in entity recognition accuracy improve the KG’s overall quality. Precise entity extraction supports reliable graph-based clinical applications downstream.

Our approach establishes a strong methodological baseline and serves as a foundational step for future enhancement. The use of a clinical knowledge base from a single medical center and website provided a high-quality, internally consistent dataset, enabling rigorous validation of our data extraction and KG construction pipeline while demonstrating its effectiveness in capturing detailed liver cancer insights. However, as real-world clinical data, our sample of 304 liver cancer patients from a single institution may introduce inherent biases. Furthermore, in broader real-world deployments, conflicting or inconsistent information across data sources is inevitable. While the current single-institution dataset largely minimized this issue, future expansions will require explicit strategies to ensure reliability.

Building on our solid proof-of-concept, the next logical steps are designed to directly address these challenges above. To enhance generalizability and mitigate bias, we will incorporate EMRs from multiple, diverse partner institutions. To handle data conflicts, we will implement explicit strategies such as source reliability weighting and expert-in-the-loop adjudication. This dual approach of expanding data diversity while ensuring its reliability is crucial for creating a comprehensive and truly trustworthy multi-layered view of liver cancer, especially as we integrate complex multi-modal data like genomic information (Eralp and Sefer, 2024).

Similarly, our current knowledge fusion process, which leverages TF-IDF, proved highly effective for rapid and reliable lexical entity normalization. To further elevate the graph’s semantic intelligence, we plan to replace TF-IDF with large language model-based contextual embedding to enable more nuanced entity linking and knowledge integration (Yang et al., 2024). These models demonstrate a superior ability to understand the complex semantics and context of medical entities compared to traditional similarity measures.

Recognizing that the long-term value of a clinical knowledge graph depends on its ability to evolve, we have also designed a comprehensive strategy to transition our KG from a static snapshot into a dynamic clinical asset. Building on our proposed incremental update framework, which continuously processes new EMRs, clinical guidelines, and emerging literature (Xu et al., 2024), we will incorporate a human-in-the-loop validation workflow. This ensures that as the graph scales, its clinical accuracy and trustworthiness are maintained through expert review. This forward-looking architecture, supported by the technical scalability of our graph database, is crucial for sustained clinical relevance in a fast-moving field like oncology.

Ultimately, the goal of this work is to create a dynamic knowledge asset that can power advanced clinical decision support systems. Once enhanced with multi-modal data and deeper inference capabilities, the KG could serve as the backbone for sophisticated predictive models. For example, it could provide the structured knowledge required to apply graph neural networks for forecasting patient-specific outcomes, such as predicting drug responses via diffusion-based graph attention networks (Sefer, 2025). This bridges the gap between foundational knowledge representation and actionable, personalized medicine.

## 6 Conclusion

This study’s key contribution is the creation of a workflow that extracts KGs from Chinese EMRs, aiming to support the development and application of Traditional Chinese Medicine KGs in disease diagnosis and treatment. In this study, the conceptual layer of the KG was developed based on primary liver cancer treatment guidelines and expert consultations. The DERM-RoBERTa-wwm-large-BiLSTM-CRF model was used to extract entities, including patients, examinations, symptoms, and treatments, from EMRs. The model demonstrated strong performance on the public CMeEE-v2 dataset with an F1 score of 68.84%, outperforming existing baseline models. When applied to RLC-EMRs, the proposed approach achieved a 4.3% improvement in the F1 score, along with a 5.8% increase in precision and a 3.0% enhancement in recall compared to the baseline model. Next, the entities were standardized using CCMT-2019 and combined with XYWY.com for KF. The resulting triplets were subsequently stored in the Neo4j database.

Utilizing this conceptual layer design, a KG was constructed to enable intelligent diagnosis and treatment recommendations for liver cancer. Through an evaluation of the conceptual layer design, data layer construction, and application layer functionality, the rationality, effectiveness, and practicality are validated. This study offers a framework for efficiently designing and building KGs applicable to diagnosing and treating other diseases.

Looking forward, our methodology can be generalized to other complex diseases. Future work will focus on enriching the KG with multi-center data for better representation and incorporating multi-modal information for deeper insights. By developing automated update mechanisms and integrating with advanced AI, this framework can evolve into a dynamic and truly supportive clinical knowledge resource.

## Data Availability

The raw data supporting the conclusions of this article will be made available by the authors, without undue reservation.

## Footnotes

^1^ https://tianchi.aliyun.com/dataset/95414

^2^ https://www.jinglingbiaozhu.com/

^3^ https://tianchi.aliyun.com/dataset/144495
